# Markers of imminent myocardial infarction

**DOI:** 10.1038/s44161-024-00422-2

**Published:** 2024-02-12

**Authors:** Stefan Gustafsson, Erik Lampa, Karin Jensevik Eriksson, Adam S. Butterworth, Sölve Elmståhl, Gunnar Engström, Kristian Hveem, Mattias Johansson, Arnulf Langhammer, Lars Lind, Kristi Läll, Giovanna Masala, Andres Metspalu, Conchi Moreno-Iribas, Peter M. Nilsson, Markus Perola, Birgit Simell, Hemmo Sipsma, Bjørn Olav Åsvold, Erik Ingelsson, Ulf Hammar, Andrea Ganna, Bodil Svennblad, Tove Fall, Johan Sundström

**Affiliations:** 1 Department of Medical Sciences, Uppsala University, Uppsala, Sweden; 2 Uppsala Clinical Research Center, Uppsala University, Uppsala, Sweden; 3 BHF Cardiovascular Epidemiology Unit, Department of Public Health and Primary Care, University of Cambridge, Cambridge, UK; 4 BHF Centre of Research Excellence, University of Cambridge, Cambridge, UK; 5 NIHR Blood and Transplant Research Unit in Donor Health and Genomics, University of Cambridge, Cambridge, UK; 6 HDR UK Cambridge, Wellcome Genome Campus and University of Cambridge, Cambridge, UK; 7 Department of Clinical Sciences, Skåne University Hospital, Lund University, Malmö, Sweden; 8 K.G. Jebsen Center for Genetic Epidemiology, Department of Public Health and Nursing, NTNU, Norwegian University of Science and Technology, Trondheim, Norway; 9 HUNT Research Center, Department of Public Health and Nursing, NTNU, Norwegian University of Science and Technology, Levanger, Norway; 10 Genomic Epidemiology Branch, International Agency for Research on Cancer (IARC/WHO), Lyon, France; 11 Levanger Hospital, Nord-Trøndelag Hospital Trust, Levanger, Norway; 12 Estonian Genome Centre, Institute of Genomics, University of Tartu, Tartu, Estonia; 13 Clinical Epidemiology Unit, Institute for Cancer Research, Prevention and Clinical Network (ISPRO), Florence, Italy; 14 Navarra Public Health Institute, Pamplona, Spain; 15 IdiSNA, Navarra Institute for Health Research, Pamplona, Spain; 16 Finnish Institute for Health and Welfare, Helsinki, Finland; 17 Lifelines Cohort Study, Groningen, Netherlands; 18 Department of Endocrinology, Clinic of Medicine, St. Olavs Hospital, Trondheim University Hospital, Trondheim, Norway; 19 Science for Life Laboratory, Uppsala University, Uppsala, Sweden; 20 Institute for Molecular Medicine Finland, University of Helsinki, Helsinki, Finland; 21 Analytic and Translational Genetics Unit, Massachusetts General Hospital, Boston, MA USA; 22 Stanley Center for Psychiatric Research, Broad Institute of MIT and Harvard, Cambridge, MA USA; 23 Department of Surgical Sciences, Uppsala University, Uppsala, Sweden; 24 The George Institute for Global Health, University of New South Wales, Sydney, New South Wales Australia

**Keywords:** Prognostic markers, Myocardial infarction

## Abstract

Myocardial infarction is a leading cause of death globally but is notoriously difficult to predict. We aimed to identify biomarkers of an imminent first myocardial infarction and design relevant prediction models. Here, we constructed a new case–cohort consortium of 2,018 persons without prior cardiovascular disease from six European cohorts, among whom 420 developed a first myocardial infarction within 6 months after the baseline blood draw. We analyzed 817 proteins and 1,025 metabolites in biobanked blood and 16 clinical variables. Forty-eight proteins, 43 metabolites, age, sex and systolic blood pressure were associated with the risk of an imminent first myocardial infarction. Brain natriuretic peptide was most consistently associated with the risk of imminent myocardial infarction. Using clinically readily available variables, we devised a prediction model for an imminent first myocardial infarction for clinical use in the general population, with good discriminatory performance and potential for motivating primary prevention efforts.

## Abstract (web summary)

Gustafsson, Lampa et al. used proteomics, metabolomics and clinical factors in a case–cohort study to identify biomarkers of imminent myocardial infarction and devise a prediction model for imminent myocardial infarction, which can be clinically used in the general population.

## Main

Despite declining age-standardized rates, myocardial infarction remains the leading and increasing cause of death globally^1^. Prevention of myocardial infarction is highly prioritized^2^, but the targeting of primary preventive efforts is hampered by inefficient means of identifying individuals at the highest risk for an imminent myocardial infarction (IMI). This could be partially explained by the inability of most risk prediction models to account for the highly dynamic nature of the period leading up to a myocardial infarction. For instance, traumatic events, such as a cancer diagnosis or loss of a spouse, markedly increase the risk of myocardial infarction^3,4^. In addition, the degree of stenosis in the culprit lesion in the coronary artery appears to increase in the months just before the myocardial infarction^5^. Nonetheless, to date, most biomarkers have been investigated over several years of follow-up because of a low number of individuals with a first myocardial infarction shortly after baseline in the general population. Hence, a large population-based study focusing on identifying biomarkers of an IMI is needed.

Primary prevention for asymptomatic risk factors over a long period is costly, and motivation among patients and providers is limited even for secondary prevention^6^. Risk prediction in the short term based on biomarkers of IMI might tilt the scales for prevention, as the knowledge of an increased risk of a first myocardial infarction within the ensuing few months might motivate patients and doctors to consider preventive strategies.

We hypothesized that circulating biomarkers of the dynamic biological processes that operate in the months preceding a myocardial infarction could be measured and used to assess risk. We tested this in a new nested case–cohort study and devised a prediction model for an imminent first myocardial infarction.

## Results

**Fig. 1:**
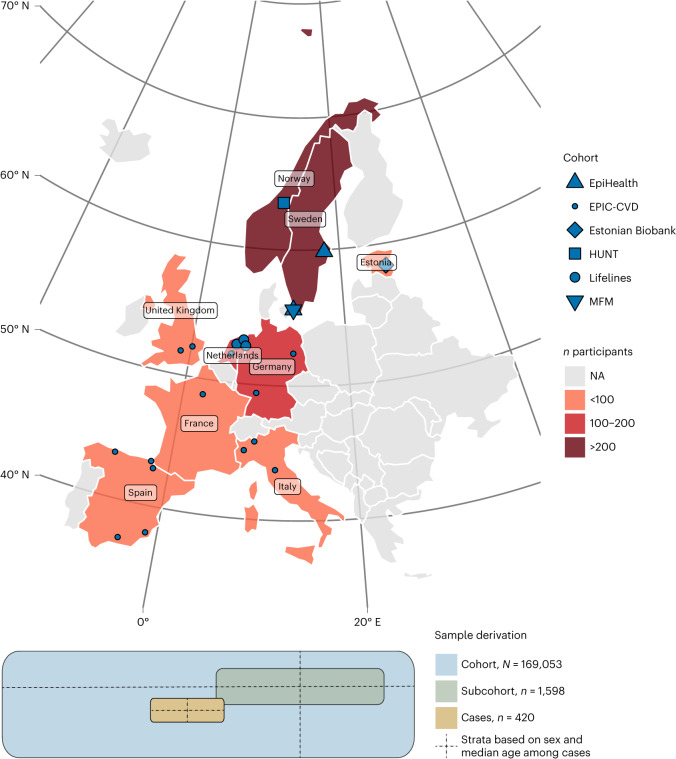
Derivation of the sample representing 169,053 individuals without previous cardiovascular disease from six European population-based cohorts. The distribution of MIMI participants across Europe is shown, with the participating countries and cohort centers indicated. Cases (*n* = 420) were initially sampled, and center-specific strata based on sex and median age were constructed. From each cohort center, up to four subcohort representatives were drawn for each case from the same stratum. A subcohort (*n* = 1,598) weighted to represent the total cohort (*N* = 169,053) based on the number of individuals in the age and sex strata in the total cohort was thus assembled. NA, not applicable.

We assembled a new nested case–cohort study, the Markers of Imminent Myocardial Infarction (MIMI) study. The study includes initially cardiovascular disease-free individuals in six European general population-based cohorts who developed a myocardial infarction within the first 6 months after the baseline examination, with up to four cohort representatives per case (Fig. 1 and Supplementary Table 1). The case–cohort design allows for time-to-event analyses and derivation of accurate prediction models; it is also less prone to certain biases than the case–control design^7^. After exclusions, data of 2,018 individuals weighted to represent the full cohort of 169,053 persons were available for analysis (420 IMI cases and 1,598 subcohort representatives). Their characteristics at baseline are shown in Extended Data Table 1.

Thereafter, we determined the levels of 817 proteins (some duplicates) and 1,025 metabolites in biobanked plasma samples from the cohort baseline examinations in a core laboratory and harmonized 16 clinical variables between the cohorts. We divided the study sample into a discovery sample (EpiHealth, Trøndelag Health Study (HUNT) and Lifelines; 70% of the sample) and an external validation sample (European Prospective Investigation into Cancer and Nutrition—Cardiovascular Disease (EPIC-CVD), Estonian Biobank study and Malmö Preventive Project (MFM); 30% of the sample). Considering the limited sample size of the study, we also performed an internal validation as an exploratory analysis by randomly splitting the study sample into a 70:30 discovery/validation sample, repeated in 100 random draws.

We investigated the associations of proteins, metabolites and clinical variables with the risk of a first myocardial infarction within 6 months after baseline using weighted, stratified Cox proportional-hazards regression models in the discovery sample. Biomarkers that passed multiple testing bounds (a Benjamini–Hochberg false discovery rate (FDR) of <0.05) were verified in the same models in the validation sample (this was done in the external and internal validation sets), with directionally consistent results at *P* < 0.05 considered replicated.

**Fig. 2:**
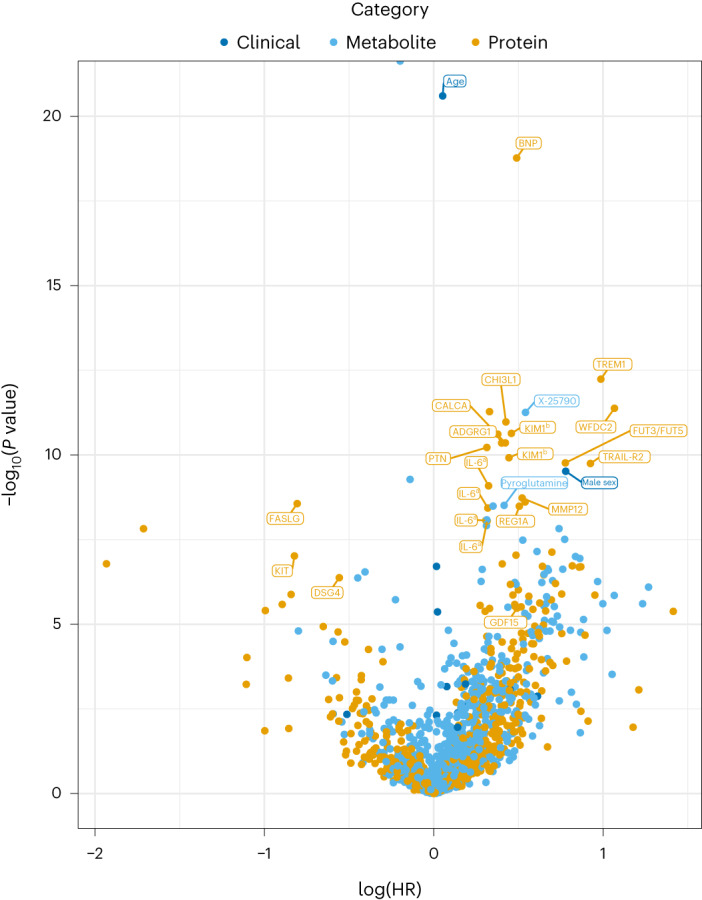
Associations of proteins, metabolites and clinical variables with IMI risk. The associations of 817 proteins, 1,025 metabolites and 16 clinical variables with the risk of a first myocardial infarction within 6 months in the full MIMI study, adjusted for technical covariates, are shown by biomarker category (clinical, metabolite or protein). HR relates to a doubling of the concentration of proteins and metabolites and a one-unit higher level of clinical biomarkers on their original scale (for example, years, mmol l^−1^). The top 25 biomarkers that passed external validation and ranked on how many internal validation splits the biomarker passed the replication criteria in the model adjusted for technical covariates in addition to the external validation are highlighted. ^a^IL-6 and ^b^KIM1 were measured on multiple Olink panels and tested in separate statistical tests. *n* = 420 cases and 1,598 noncases.

**Fig. 3:**
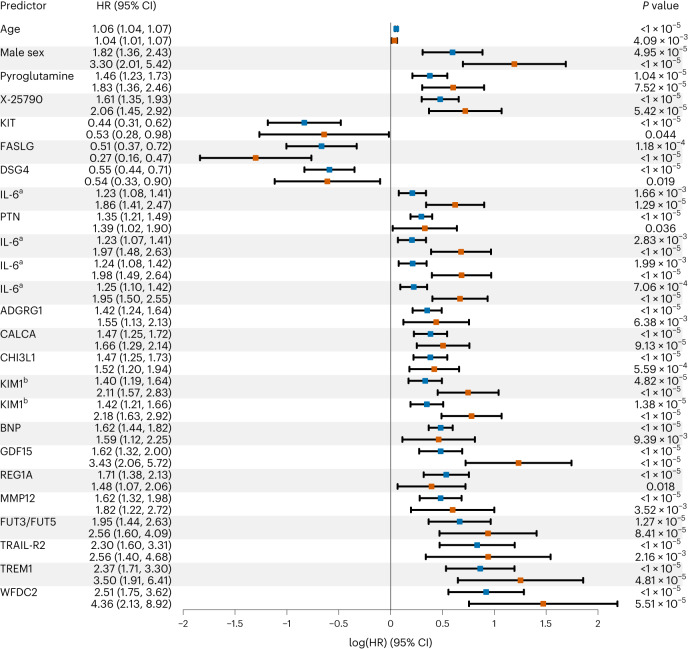
Top variables associated with IMI risk. The top 25 biomarkers that passed external validation and ranked on how many internal validation splits the biomarker passed the replication criteria in the model adjusted for technical covariates in addition to the external validation are shown. Each predictor is represented by two rows, with the discovery result (blue) presented first and the validation result presented second (red). The results are sorted by predictor type (clinical, metabolite or protein) and effect size from the combined analysis of the discovery and validation samples. *P* value was calculated based on a 2 d.f. Wald test for metabolites analyzed using the missing indicator method (biomarker and missing indicator) and a 1 d.f. Wald test otherwise (biomarker only), two-sided in both cases. The 95% CI of the point estimate (log(HR)) was calculated for the biomarker only and might include 1 even if *P* < 0.05 from the 2 d.f. (biomarker + indicator) Wald test. ^a^IL-6 and ^b^KIM1 values were determined from multiple Olink panels and tested in separate statistical tests. *n* = 296 cases and 1,121 noncases in the discovery sample; *n* = 124 cases and 477 noncases in the validation sample.

In one-by-one models adjusting for technical covariates (season, storage time and plate; Fig. 2), 48 proteins, 43 metabolites and 3 clinical variables (age, sex and systolic blood pressure) were found to be associated with IMI after the discovery–validation process (Fig. 3 and Supplementary Table 2).

Thereafter, we investigated promising markers in models further adjusting for age and sex. Among them, brain natriuretic peptide (BNP) was the only biomarker with a borderline significant association with IMI (HR per doubling of BNP level (95% confidence interval (95% CI)) = 1.33 (1.15, 1.55), *P* = 1.63 × 10^−4^, FDR = 0.11 in the discovery sample and 1.40 (1.00, 1.94), *P* = 0.049 in the validation sample; Extended Data Fig. 2). BNP was the only biomarker with a suggestive association in the internal validation, passing the formal replication criteria in 22 of 100 random splits. By comparison, stem cell factor (SCF) and interleukin-6 (IL-6), biomarkers with a weaker support of an association, replicated in only 5 or 4 of 100 random splits. The cumulative hazard of IMI by fourths of BNP is shown in Extended Data Fig. 3. The associations of BNP with IMI in sensitivity analyses excluding one cohort at a time and in a random-effects meta-analysis were similar, as shown in Extended Data Figs. 4 and 5. For some of the 94 variables, we observed substantial between-cohort heterogeneity in the estimates when they were evaluated in a random-effects meta-analysis (Supplementary Table 3). The addition of interaction terms between sex and the biomarkers did not reveal any additional associations. Associations with IMI within 3 months (185 cases) were similar to those within 6 months (Extended Data Fig. 6).

**Extended Data Fig. 1:**
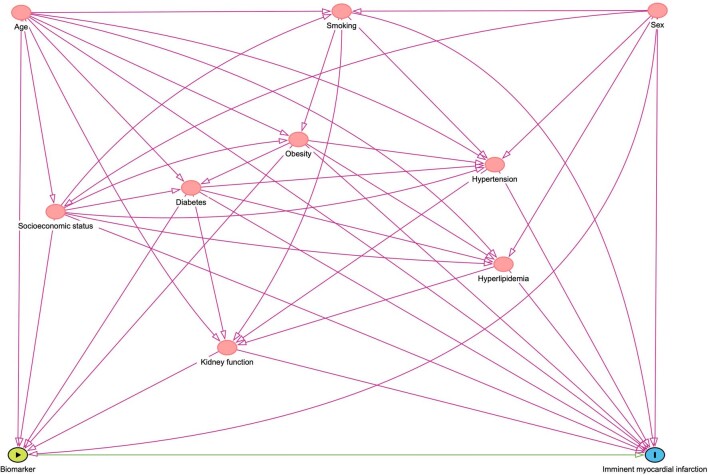
Causal assumptions for developing bias- minimized models. Directional acyclic graph (DAG) showing the best-guess relationship between the exposure (BNP) and the outcome (IMI) and other factors influencing this relationship together with the expected direction.

**Extended Data Fig. 2:**
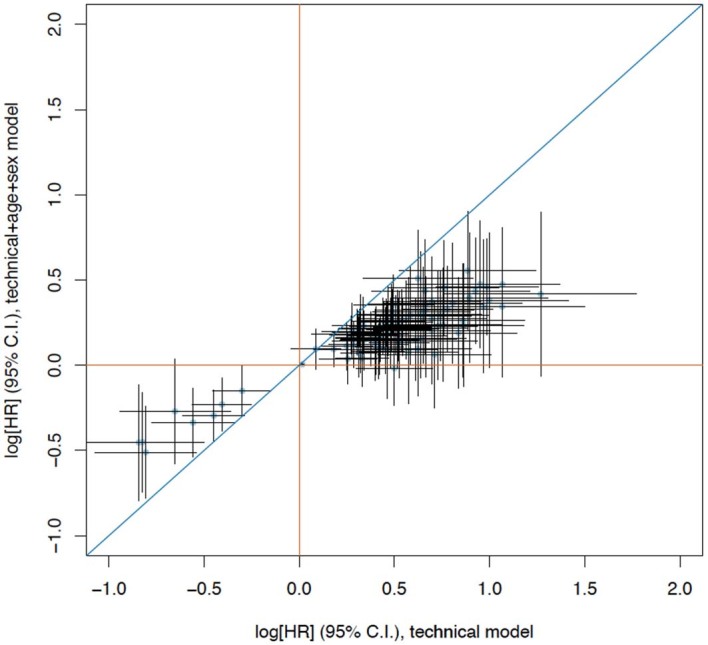
Variables associated with risk of an imminent myocardial infarction, further adjusted for age and sex. Scatter plot comparing the point estimate (log[HR]) and corresponding 95% confidence interval from models adjusting for technical covariates (x-axis) and models additionally adjusting for age and sex (y-axis) in the full MIMI study. The 95% C.I. is calculated for the biomarker only whereas the p-value in the tables is based on the 2 d.f. Wald test (two-sided) of biomarker and missing indicator (when used), hence the 95% C.I. might overlap with null for a p-value < 0.05. N=420 cases and 1598 non-cases.

**Extended Data Fig. 3:**
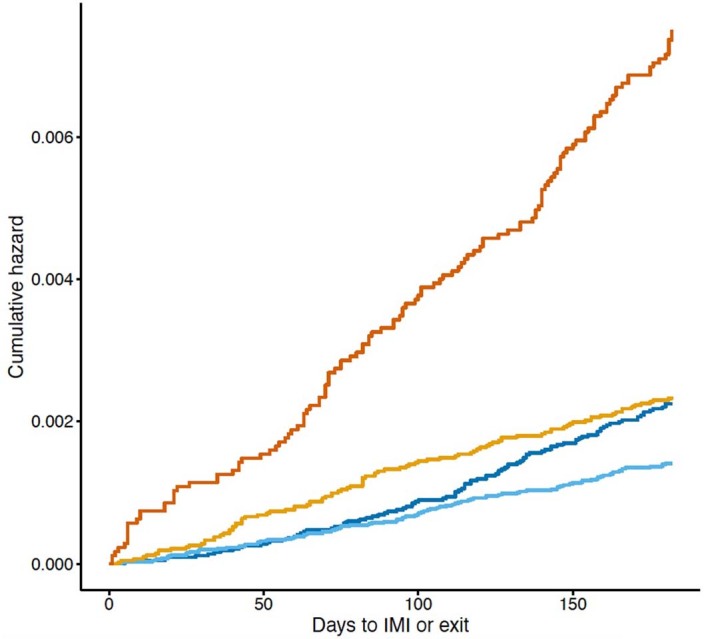
Association of BNP with Risk of an Imminent Myocardial Infarction. Kaplan–Meier graph of unadjusted cumulative hazard of an imminent myocardial infarction (IMI) by fourths (Q) of brain natriuretic peptide (BNP), weighted by sampling weights.

**Extended Data Fig. 4:**
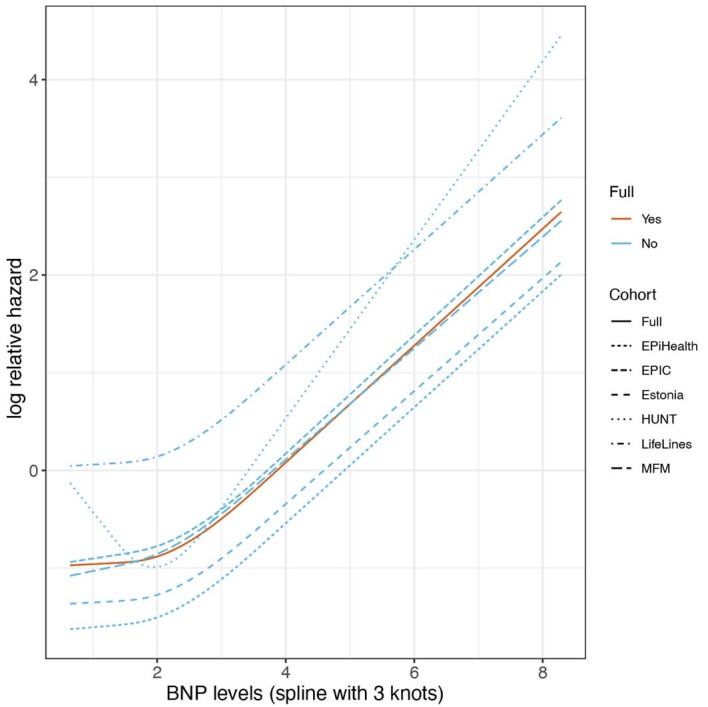
Leave-one-out analyses of the association of BNP with imminent myocardial infarction. Model with BNP and technical covariates in leave-one-out analyses where one cohort was omitted at time.

In a model investigating the total effect of the BNP–IMI association (with a priori selected confounders, not mediators, according to Extended Data Fig. 1), adjusting for age, sex, weight, height, creatinine and systolic blood pressure, the association of BNP with IMI remained similar (HR (95% CI) = 1.34 (1.14, 1.57), *P* = 3.12 × 10^−4^ in the discovery sample and 1.51 (1.05, 2.18), *P* = 0.028 in the validation sample; per doubling of BNP level).

We then investigated the association of the most promising marker, BNP, with the coronary artery calcium score (CACS) at a cardiac computer tomography examination in an external population-based cohort of 1,586 participants of the Swedish CArdioPulmonary bioImage Study (SCAPIS) who were free from self-reported cardiovascular disease. Here, a higher CACS was not notably associated with a higher BNP level (odds ratio (95% CI) = 1.14 (0.91, 1.42), *P* = 0.25; per doubling of BNP level) in an ordinal regression model adjusting for the same covariates as in the total-effects model.

**Fig. 4:**
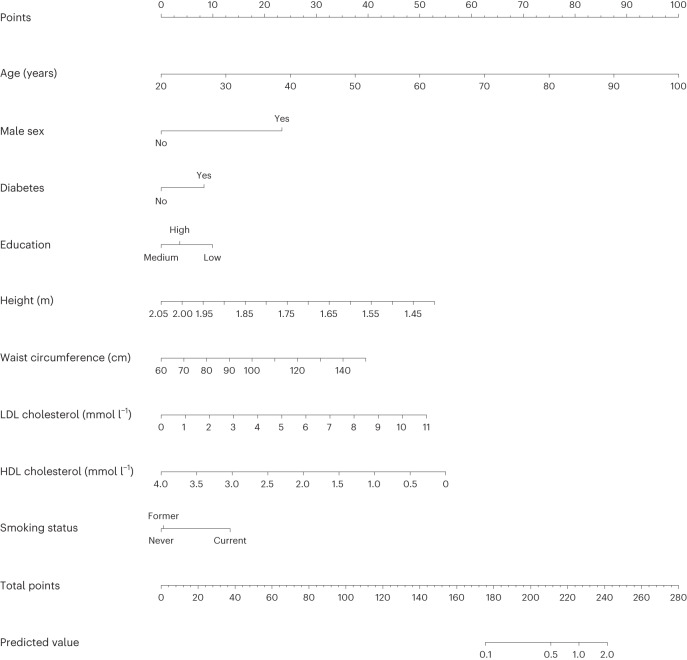
Nomogram of the model for the clinical prediction of an IMI. A nomogram for predicting IMI risk based on the final clinical model is shown. Each variable value contributes points (ruler at the top) that are summed up and translated to the predicted risk of a myocardial infarction within 6 months (bottom two rulers). Equation, *β* coefficients, 6-month survival and mean variable values are provided in Supplementary Table 4. A worked example is shown in Extended Data Fig. 8. The model is also presented as an interactive web application at miscore.org.

Finally, we investigated the possibility of developing a clinical risk prediction algorithm for a first IMI using clinically available variables and a weighted Cox ridge regression model. The prediction model achieved an internally validated C-index of 0.78, indicating a good ability to discriminate between IMI cases and noncases. When validating the model in the UK Biobank, a C-index of 0.82 was obtained, while a calibration plot showed some overestimation of 6-month IMI risks. As a comparison, the recalibrated SCORE2 achieved C-indexes of 0.77 (MIMI cohort) and 0.81 (UK Biobank) and overestimated the IMI risks in both samples (Extended Data Fig. 7). A nomogram based on the model is shown in Fig. 4, with a worked example of its intended use displayed in Extended Data Fig. 8 and its cross-validated calibration presented in Extended Data Fig. 7. An interactive web application is presented at miscore.org. Coefficients for predicting IMI from the model are shown in Supplementary Table 4.

No biomarkers improved risk prediction in a LASSO (least absolute shrinkage and selection operator) Cox regression model; the variable selection by the LASSO was unstable, with the 95% bootstrap CI on the model size being 0–128 variables. No biomarkers improved risk prediction in a random forest model using 2,000 trees; it also ranked BNP and N-terminal pro-BNP (NT-proBNP) at the top but with very large CIs (Supplementary Table 5).

## Discussion

We here set out to identify and test biomarkers and the predictability of an imminent first myocardial infarction using a new case–cohort consortium of individuals without prior cardiovascular disease and with biobanked blood samples. From more than 1,800 biomarkers, we identified 48 proteins, 43 metabolites and 3 clinical variables associated with the risk of an imminent first myocardial infarction independent of technical covariates. Further analyses revealed BNP as the only biomarker consistently associated with IMI risk. We also derived a prediction model to discriminate between subsequent cases and noncases. The IMI phenotype has rarely been studied prospectively in the general population and with a broad panel of biomarkers. The findings may have implications for both clinical primary prevention studies and further etiological studies.

**Extended Data Fig. 5:**
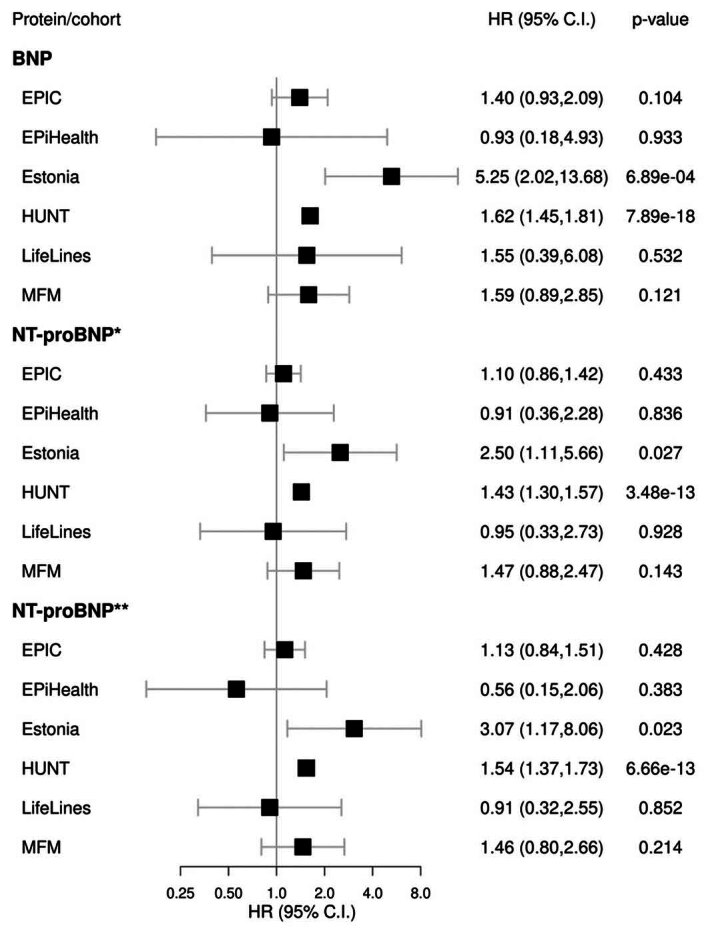
Associations of BNP and NT-proBNP with imminent myocardial infarction in the individual cohorts. Forest plot of the regression results in the model adjusting for technical covariates performed per cohort for all available BNP measurements (BNP and N-terminal pro form). Hazard ratio and corresponding 95% confidence interval is presented. NT-proBNP is measured on both the CVD2 (*) and Metabolism panel (**). Individual cohort sample sizes are given in Supplementary Table 1.

**Extended Data Fig. 6:**
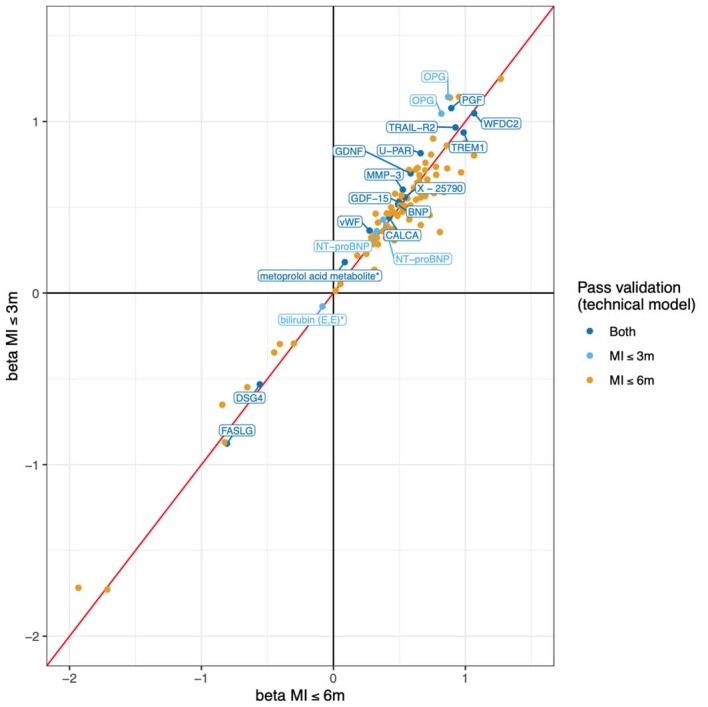
Comparison of associations of biomarkers with risk of IMI within 3 vs 6 months. Regression estimates from models with time to IMI within 3 vs 6 months as outcomes. MI, myocardial infarction.

**Extended Data Fig. 7:**
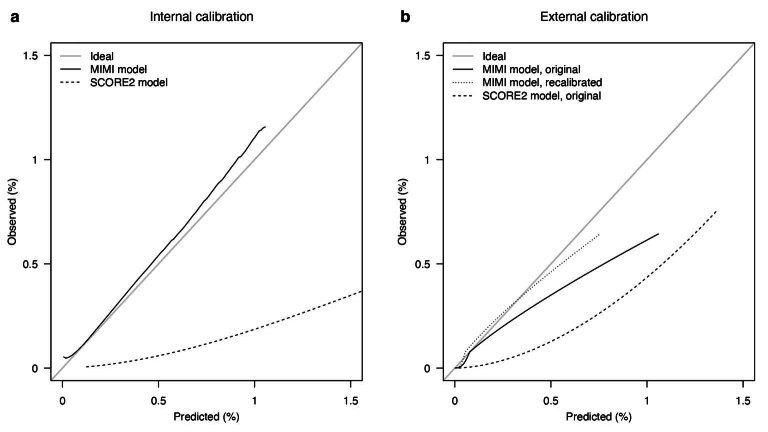
Calibration of the prediction model. **a** Internal calibration. Cross-validated calibration curves for predicted probabilities of an imminent myocardial infarction from the MIMI model (solid black line) and the SCORE2 model (dashed black line). **b** External calibration. Calibration curves for predicted probabilities of an imminent myocardial infarction from the original MIMI model (solid black line), the MIMI model recalibrated to the UKBB data (dotted black line), and the original SCORE2 model (dashed black line). The diagonal gray line in both panels is the line of ideal calibration where predicted probabilities match the observed fraction experiencing the event.

**Extended Data Fig. 8:**
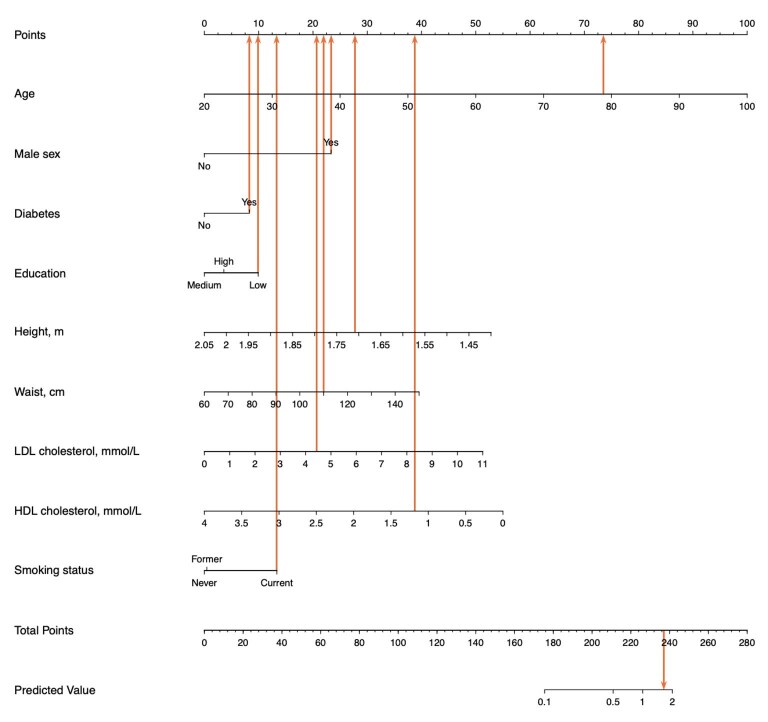
Worked example of nomogram use. A 78-year-old (73 points) smoking (13 points) low-educated (10 points) man (23 points) with diabetes (8 points), height 1.71 m (28 points), waist circumference 110 cm (22 points), LDL cholesterol 4.5 mmol/L (21 points) and HDL cholesterol 1.2 mmol/L (39 points) will have a total score of 73 + 13 + 10 + 23 + 8 + 28 + 22 + 21 + 39 = 237 points, corresponding to a 6- month risk of a first myocardial infarction of circa 1.58%. The model is also presented as an interactive web application on miscore.org.

In the current study, higher BNP levels in individuals without a known cardiovascular disease were linked to a higher risk of a first myocardial infarction within 6 months in several models. Cardiomyocytes produce BNP in response to strain^8^, and NT-proBNP measurement is a pillar of the clinical management of heart failure^9^ but is not used in diagnosing myocardial infarction^10^. Diastolic dysfunction is an early feature of myocardial ischemia, and a higher BNP level in this context is likely underpinned by diastolic dysfunction caused by subclinical ischemia^11^ in individuals with some degree of coronary stenosis. This is supported by the weak association of BNP and CACS observed herein, although the association should be interpreted carefully. The noncausal explanation is further supported by the noncausality suggested by Mendelian randomization studies (acknowledging that associations of genetically determined lifelong BNP levels with coronary disease may have limited relevance to a temporally boxed-in series of events): a genetic variant affecting the expression of the BNP gene (*NPPB*, rs198389) is not associated with cardiovascular endpoints^12^ or coronary artery disease^13^. The influence of chance on the finding is low, as NT-proBNP was also significantly associated with IMI in the discovery sample, with a borderline association in the validation sample (Extended Data Fig. 5). While BNP may hence reflect an underlying coronary artery disease, it did not add materially to a risk prediction model for IMI composed of more readily available biomarkers.

Several known mechanisms implicated in atherosclerosis and ischemia were represented among the other 94 biomarkers associated with an IMI in both the discovery and validation samples after adjusting for technical covariates, including inflammation (IL-6)^14^, extracellular matrix metabolism (WAP four-disulfide core domain protein 2 (WFDC2))^15^, hypertrophy (adhesion G-protein-coupled receptor G1 (AGRG1))^16^, apoptosis (triggering receptor expressed on myeloid cells 1 (TREM1), tumor necrosis factor receptor superfamily member 10B (TRAIL-R2)) and cell adhesion (AGRG1). We also observed associations with markers representing mechanisms less often implicated in coronary diseases, such as markers of kidney injury (kidney injury molecule 1 (KIM1))^17^, appetite regulation (growth differentiation factor 15 (GDF15))^18^, and an α-amino acid found in dietary supplements and associated with paracetamol use (pyroglutamine)^19^. While some associations may be causal, others, such as associations with levels of chitinase-3-like protein 1 (CHI3L1)^20^, pleiotrophin (PTN) or KIT, may more likely be responses to myocardial ischemia. These findings may accelerate further etiological studies of acute coronary events.

We here developed a prediction model for IMI in the general population. An imminent infarction is difficult to predict; the signals are weak, and we faced power limitations. The model achieved good discriminative ability, with acceptable calibration in the lower risk range. It is possible to transpose to other settings by entering the base hazards and variable means of those settings, for example, interactively at miscore.org. Given the increasing global burden of deaths from myocardial infarction, the importance of predicting them and increasing the individual motivation for preventing such deaths may be substantial; this can be tested in clinical trials.

The current study has several limitations. First, the use of multiple cohorts introduced heterogeneity. We addressed this at the sampling, biomarker analysis and statistical analysis stages, with the resulting limitation that the heterogeneity decreases statistical power. The strengths are the same as in other multicenter studies, including that only biomarkers with consistent importance in different settings are brought forward. Other study limitations are inherent to the uncertainty of ranking the top findings and the inability of one-by-one strategies to capture complex interrelationships. The instability of the variable importances from the random forest was unsurprising, as such methods are notoriously data hungry and require far larger datasets than classical modeling techniques^21^. While the studied markers are easily obtainable by a simple blood test or clinical assessment, a limitation is that a blood sample will not always capture tissue-specific processes. In addition, our study was limited to proteins and metabolites that remain stable in the freezer for many years. The biomarker analyses used herein are currently not available in clinical practice, and we lacked the clinically available and more precise immunoassay measurements of, for example, NT-proBNP and cardiac troponin; hence, imprecision in the proximity extension assay and ultra-high performance liquid chromatography–mass spectrometry (UPLC–MS) technologies may preclude definitive mechanistic insights and maximal clinical utility. Further, making causal assumptions is fundamentally challenging in a multimarker landscape where many causal pathways are unknown. Most markers could be potential mediators in pathways for known causes of myocardial infarction, including age and sex. Consequently, we provided models adjusted for technical covariates only and models with further biological covariate adjustment. Thus, some associations could be explained by confounding by, for example, age and sex. Notably, mediators of causal effects are also important to identify, with implications for prediction and use as treatment targets.

In conclusion, we identified biomarkers associated with the risk of an imminent first myocardial infarction, including BNP. Delineation of the distinct biological processes that operate in the months before the first myocardial infarction will be key to discovering prevention targets. We developed and validated a prediction model with a fair ability to discriminate between persons with and without risk of an imminent first myocardial infarction. Risk prediction in the short term may enhance the motivation of patients and doctors for primary prevention.

## Methods

### Study sample and outcome

The MIMI study sample draws biobanked blood and data from six European cohorts of the BBMRI-LPC (Biobanking and Biomolecular Research Infrastructure—Large Prospective Cohorts) collaboration^22^, as shown in Fig. 1 and Supplementary Table 1. After sample size determination, we supplied each cohort with a standardized protocol (in which all definitions are described in detail) and an R script for selecting cohort representatives for the subcohort (Supplementary Notes).

Cohort participants with biobanked samples (at least 250 μl of plasma or serum; eventually, only plasma was included) and no previous clinical cardiovascular disease were eligible for inclusion in the present study. The exclusion criteria were previous clinical cardiovascular disease (defined as the presence at any time before baseline of any of the following: myocardial infarction, coronary procedure, heart failure, structural heart disease, tachyarrhythmias, stroke, thromboembolic disease and peripheral vascular disease) and renal failure.

Individuals with acute myocardial infarction (International Classification of Diseases, tenth revision (ICD-10), I21; ICD-9, 410.0–410.6 and 410.8) as the primary cause of hospitalization or death within 6 months after baseline were defined as IMI cases. We included both ST-elevation and non-ST-elevation myocardial infarctions; we encouraged efforts to include only type 1 myocardial infarctions by not counting cases with any of the following ICD codes in secondary positions: anemia (for example, ICD-10, D50–D64; ICD-9, 280–285), tachyarrhythmias (for example, ICD-10, I47–I49; ICD-9, 427), heart failure (for example, ICD-10, I50; ICD-9, 428), renal failure (for example, ICD-10, N17–N19; ICD-9, 584–586), chronic obstructive pulmonary disease (for example, ICD-10, J43–J44; ICD-9, 491, 492 and 496), sepsis and other severe infections (for example, ICD-10, A40–A41; ICD-9, 038), or hypertensive crises.

Up to four cohort representatives per available IMI case were randomly drawn from the full cohort to the subcohort in 50 strata based on sex, age (above/below median) and study center in a stratified case–cohort design^7^. All 420 IMI cases, and 1,598 subcohort representatives, were drawn from the full cohort of 169,053 participants, as summarized in Fig. 1.

### Exposures

Clinical variables (age, sex, height, weight, waist circumference, systolic and diastolic blood pressure, triglycerides, high-density lipoprotein (HDL) cholesterol, non-HDL cholesterol, low-density lipoprotein (LDL) cholesterol, total cholesterol, glucose, diabetes status, highest education, smoking status, previous smoking exposure, alcohol intake and physical activity) were harmonized between the cohorts (Supplementary Notes). Non-HDL cholesterol was calculated as total cholesterol − HDL cholesterol. LDL levels were calculated using the extended Martin–Hopkins equation^23^.

All blood samples were randomized into appropriate measurement plates, stratified by cohort (with a similar number from each cohort on every plate), and aliquoted into the plates. Quality controls are summarized below and described in detail in the Supplementary Notes.

Protein measurements were done using the Olink proximity extension assay (Olink), a highly specific 92-plex immunoassay. Overall, 829 proteins across nine panels (cardiometabolic, cardiovascular II, cardiovascular III, development, immune response, inflammation, metabolism, oncology II and organ damage) were analyzed, including 804 unique proteins (considering overlap between panels). Relative protein values on a log_2_ scale are reported, with each protein value normalized by plate by centering all plates at the same median, assuming random plate placement. Values below the assay’s lower limit of detection (LOD) were also included in the analyses.

Metabolites were analyzed using the UPLC–tandem MS (UPLC–MS/MS)-based Metabolon platform (Metabolon) by four different methods: reversed-phase UPLC–MS/MS with positive-mode electrospray ionization (early and late phase), reversed-phase UPLC–MS/MS with negative-mode electrospray ionization, and hydrophilic interaction LC/UPLC–MS/MS with negative-mode electrospray ionization. Overall, 1,135 metabolites were captured, including 925 with known identity and 210 with unknown identity. Relative metabolite levels were determined and normalized by analysis day. Metabolite levels were log_2_ transformed, and nondetectable levels (<LOD or metabolite not present in the sample) were constant value imputed to a value below the minimum metabolite value (minimum/sqrt(2)).

Samples that did not satisfy the quality control criteria were initially excluded; exclusion filters were applied separately for the proteomics and metabolomics analyses, and only samples passing quality control for both analyses were included in the analysis set. For the proteomics analysis, samples with more than 50% of panels failing for technical reasons were excluded (*n* excluded = 33). For the metabolomics analysis, samples were excluded because of low volume or detection of fewer metabolites than expected (*n* excluded = 4). Consequently, samples for 420 cases and 1,598 subcohort representatives remained for analysis.

Next, biomarkers with an extremely high proportion of nondetectable or below-LOD measurements were excluded, with the same exclusion filters for proteins and metabolites. Biomarkers had to be detected in all six cohorts with at least 30 detectable values across all cohorts (~1.5% of the MIMI samples) or were otherwise excluded. Consequently, 817 proteins (some duplicates) and 1,025 metabolites were retained for analysis.

### Statistical analysis

All analyses were done using R (version 4.1.1)^24^ with the glmnet^25^, mice^26^, rms^27^, ranger^28^ and survival^29^ add-on packages.

#### One-by-one etiological analyses

In the discovery sample, the associations of all clinical variables (listed in Extended Data Table 1), proteins and metabolites with IMI were analyzed in separate weighted, stratified Cox proportional-hazards regression models adjusting for covariates, as described below. Inverse sampling probability weights (Borgan II) were applied to account for the case–cohort design in a stratified model, allowing for a different shape of the baseline hazard for each MIMI cohort (six levels) and using a robust variance estimator (Huber–White). Nonlinear relationships between continuous covariates (not including the biomarkers) and IMI were modeled using restricted cubic splines, and all factor variables were considered unordered.

Associations with an FDR (Benjamini–Hochberg) of <0.05 were taken forward to the validation sample, in which directionally consistent results with *P* < 0.05 were considered replicated.

Cox proportional-hazards models adjusting for technical covariates (season, storage time and plate) were initially applied. Replicating biomarkers from the model adjusting for technical covariates were investigated in a model further adjusting for age and sex. A model allowing for an interaction between the biomarker and sex was further tested. Replicating biomarkers in the model adjusted for age and sex were then subjected to causal assumptions (Extended Data Fig. 1), and a bias-minimized model for each biomarker was investigated, estimating the total effects (including the effects of mediators).

#### Missingness and sensitivity analyses

Clinical variables with high missingness (previous smoking exposure, alcohol intake and physical activity) were not used in the analyses. Protein values below the LOD were included in the analyses; nondetectable metabolite levels were replaced with a constant value, and a missing indicator was added, as described below. The remaining missing values in the covariates were multiple imputed (*n* imputations = 20) using chained equations including the outcome, clinical covariates and other variables correlated with the variable in the imputation model^30^. Regression results across imputed datasets were combined using Rubin’s rules^31^.

Interactions with sex were investigated by analyzing an interaction term for sex and each biomarker in models adjusting for technical covariates, age and sex. The interaction terms and all terms including the biomarker were tested using a multivariable chi-squared test with the same multiple-testing correction described above, requiring directionally consistent discovery and validation results.

The following secondary sensitivity analyses were included: random-effects inverse variance-weighted meta-analyses (DerSimonian–Laird) combining per-cohort results, leave-one-out analyses investigating the influence of single cohorts, complete-case analyses not imputing missing values in the clinical covariates, and analyses limiting the follow-up time to 3 months.

#### Simultaneous modeling and development of a prediction model

To attempt predicting this phenotype, we developed a prediction model for IMI using age, sex, anthropometric variables (height, weight and waist circumference), variables routinely collected in the laboratory (LDL cholesterol, HDL cholesterol, creatinine, glucose and triglycerides), systolic and diastolic blood pressure, smoking status (never, former or current) and education level. Regression coefficients were estimated using a weighted Cox ridge regression model, which shrinks coefficients toward zero using an *L*_2_ penalty to accommodate overfitting. The strength of the penalty (lambda) was determined using tenfold cross-validation over a grid of 250 lambda values, repeated 100 times. The lambda selection was repeated in each imputed dataset, and the coefficients associated with the lambda giving the lowest cross-validated deviance were extracted. The final coefficient set was obtained by taking the median of the coefficients from each imputed dataset. A single-imputed dataset was used for validation and calibration. The C-index, which indicates a model’s ability to rank the risks, was determined using 100 repeats of tenfold cross-validation. A calibration curve was constructed using 100 repeats of tenfold cross-validation^32^. All modeling steps were repeated in each fold to assess the calibration accuracy objectively. The model containing only clinical variables was then reduced by approximating the linear predictor from the full model through stepwise regression. Predictions from the full model were used as the outcome in a linear model wherein variables were dropped sequentially until *R*^2^ > 0.95. This yielded a highly parsimonious final model incorporating the main drivers of predictions. The prediction model was compared to SCORE2, a validated prediction model for the 10-year risk of cardiovascular disease developed using multiple European cohorts^33^. The 10-year survival probability and the covariate mean values used in the SCORE2 equations were replaced with the estimated 6-month survival probability and mean values from the current data to calculate the SCORE2-estimated 6-month cardiovascular disease risk^34^. Two additional external validations of the model were performed in the UK Biobank. First, all coefficients and covariate mean values in Supplementary Table 4 were used to validate the model. Second, the model was recalibrated using mean values and the estimated baseline risk from the UK Biobank cohort before validation.

To evaluate whether any biomarkers added to the clinical prediction model improve risk prediction, we used the linear predictor from the prediction model as an offset in a LASSO Cox regression model. Before model fitting, all proteins and metabolites were adjusted for technical variables. Briefly, each biomarker was used as the outcome variable in a regression model with all technical variables as covariates. The residuals from these models were used in place of the original biomarker values in the LASSO model. The LASSO model fitting was bootstrapped 250 times to investigate the stability of the variable selection.

As the biomarkers may have nonlinear associations with the outcome and interact with one another, and prior knowledge about nonlinearities and interactions among these variables is scarce, a random forest with 2,000 trees was fitted to the data as an exploratory analysis. Briefly, the random forest fits survival trees to bootstrap data samples using a random subset of the variables in each tree, handling interactions and nonlinearities naturally. A variable importance measure is associated with each variable and calculated based on the number of splits in which a variable is involved. The random forest was bootstrapped 250 times to obtain CIs for the variable importance measures.

### Further analysis of relevant biomarkers

The associations of proteins detected using the Olink panels cardiovascular II and cardiovascular III with the CACS were available for testing in individuals free from cardiovascular disease (self-reported myocardial infarction, angina, coronary intervention, heart failure, atrial fibrillation, stroke and peripheral artery disease) for 1,586 participants at the Malmö or Uppsala centers of SCAPIS^35^. A higher CACS reflects a higher myocardial infarction risk. Proteins replicated in the primary MIMI analysis (BNP) were tested for an association with the CACS using an ordinal regression model adjusting for age, sex, body mass index, systolic blood pressure, creatinine, center, Olink plate, analysis date and season.

### Consent

This study was approved by the Uppsala Ethics Authority (Dnr 2016/197). All Estonian Biobank participants signed a broad informed consent form. The study was carried out under ethical approval 258/M-21 from the research ethics committee of the University of Tartu and data release J08 from the Estonian Biobank. The Lifelines protocol was approved by the University Medical Center Groningen medical ethical committee under number 2007/152. The study was performed in accordance with the Declaration of Helsinki. The EpiHealth study was approved by the ethics committee of Uppsala University, and all participants provided informed written consent. The MFM was approved by the previous regional research committee in Lund, Sweden (2014/643), and all participants provided informed consent. Ethical review boards of the cohorts in EPIC-CVD approved the study protocol, and all participants provided written informed consent. Participation in the HUNT study was based on informed consent, and the Data Inspectorate and the Regional Ethics Committee for Medical Research in Norway approved the study.

### Reporting summary

Further information on research design is available in the Nature Portfolio Reporting Summary linked to this article.

### Supplementary information

Supplementary Information Supplementary Notes and References.

Reporting Summary

Supplementary Tables Supplementary Tables 1–5.

## Figures and Tables

**Extended Data Table 1:**
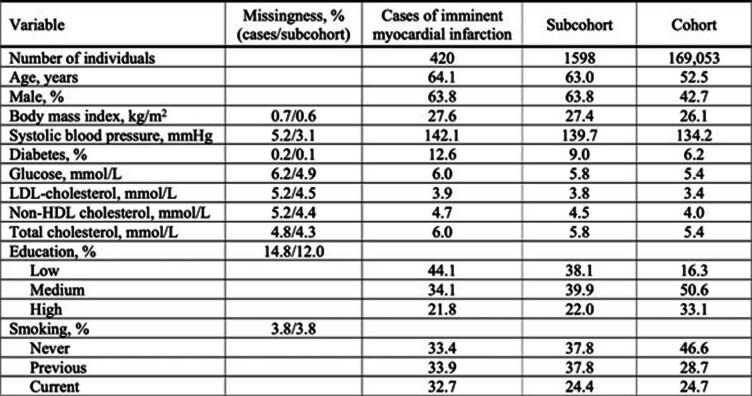
Baseline Characteristics

Baseline characteristics of the cases and weighted estimates of the full cohort of 169053 individuals from which the subcohort was drawn are shown. Numbers are percentages or arithmetic means, as indicated. LDL, low-density lipoprotein.

## Data Availability

Data supporting the findings of this study are provided in the article and related files. Raw data are not publicly available, as they contain sensitive personal information, but may be obtained from the original cohorts upon request, with varying processes, requirements and response times. For example, researchers can apply to use the Lifelines data used in this study; information on how to request access to Lifelines data and the conditions of use can be found at https://lifelines.nl/researcher/how-to-apply. Data accession codes for this study are described below.

## Code availability

The prediction model equation is available at miscore.org. Code is available at https://github.com/stefan-gustafsson-work/mimi. All code used to analyze UK Biobank data is deposited at the UK Biobank repository. Questions about the analyses and code can be sent to the corresponding author.

## References

[1] Lozano, R. et al. Global and regional mortality from 235 causes of death for 20 age groups in 1990 and 2010: a systematic analysis for the Global Burden of Disease Study 2010. *Lancet* **380**, 2095–2128 (2012). 23245604 10.1016/S0140-6736(12)61728-0 PMC10790329

[2] European Society of Cardiology, European Heart Network, European Commission, World Health Organization. European heart health charter. *Eur. J. Prev. Cardiol*. 10.1097/01.hjr.0000266926.91914.2a (2007).

[3] Carey, I. M. et al. Increased risk of acute cardiovascular events after partner bereavement: a matched cohort study. *JAMA Intern. Med.* **174**, 598–605 (2014). 24566983 10.1001/jamainternmed.2013.14558

[4] Fang, F. et al. Suicide and cardiovascular death after a cancer diagnosis. *N. Engl. J. Med.* **366**, 1310–1318 (2012). 22475594 10.1056/NEJMoa1110307

[5] Zaman, T. et al. Angiographic lesion severity and subsequent myocardial infarction. *Am. J. Cardiol.* **110**, 167–172 (2012). 22497675 10.1016/j.amjcard.2012.03.008

[6] Kotseva, K. et al. Cardiovascular prevention guidelines in daily practice: a comparison of EUROASPIRE I, II, and III surveys in eight European countries. *Lancet* **373**, 929–940 (2009). 19286092 10.1016/S0140-6736(09)60330-5

[7] Ganna, A. et al. Risk prediction measures for case–cohort and nested case–control designs: an application to cardiovascular disease. *Am. J. Epidemiol.* **175**, 715–724 (2012). 22396388 10.1093/aje/kwr374 PMC3324433

[8] Liang, F. & Gardner, D. G. Mechanical strain activates *BNP* gene transcription through a p38/NF-κB-dependent mechanism. *J. Clin. Invest.* **104**, 1603–1612 (1999). 10587524 10.1172/JCI7362 PMC409860

[9] Heidenreich, P. A. et al. 2022 AHA/ACC/HFSA guideline for the management of heart failure: a report of the American College of Cardiology/American Heart Association Joint Committee on Clinical Practice Guidelines. *Circulation* **145**, e895–e1032 (2022). 35363499 10.1161/CIR.0000000000001063

[10] Gulati, M. et al. 2021 AHA/ACC/ASE/CHEST/SAEM/SCCT/SCMR guideline for the evaluation and diagnosis of chest pain: a report of the American College of Cardiology/American Heart Association Joint Committee on Clinical Practice Guidelines. *Circulation* **144**, e368–e454 (2021). 34709879 10.1161/CIR.0000000000001029

[11] Sabatine, M. S. et al. Acute changes in circulating natriuretic peptide levels in relation to myocardial ischemia. *J. Am. Coll. Cardiol.* **44**, 1988–1995 (2004). 15542281 10.1016/j.jacc.2004.07.057

[12] Johansson, A. et al. Genome-wide association and Mendelian randomization study of NT-proBNP in patients with acute coronary syndrome. *Hum. Mol. Genet.* **25**, 1447–1456 (2016). 26908625 10.1093/hmg/ddw012

[13] Folkersen, L. et al. Genomic and drug target evaluation of 90 cardiovascular proteins in 30,931 individuals. *Nat. Metab.* **2**, 1135–1148 (2020). 33067605 10.1038/s42255-020-00287-2 PMC7611474

[14] Rai, H. et al. Association of interleukin 6 −174 G/C polymorphism with coronary artery disease and circulating IL-6 levels: a systematic review and meta-analysis. *Inflamm. Res.* **70**, 1075–1087 (2021). 34595552 10.1007/s00011-021-01505-7 PMC8572816

[15] Yamamoto, M. et al. HE4 predicts progressive fibrosis and cardiovascular events in patients with dilated cardiomyopathy. *J. Am. Heart Assoc.* **10**, e021069 (2021). 34320813 10.1161/JAHA.120.021069 PMC8475713

[16] Zhang, Y., Si, Y., Ma, N. & Mei, J. The RNA-binding protein PCBP2 inhibits Ang II-induced hypertrophy of cardiomyocytes though promoting GPR56 mRNA degeneration. *Biochem. Biophys. Res. Commun.* **464**, 679–684 (2015). 26116532 10.1016/j.bbrc.2015.06.139

[17] McCarthy, C. P. et al. Derivation and external validation of a high-sensitivity cardiac troponin-based proteomic model to predict the presence of obstructive coronary artery disease. *J. Am. Heart Assoc.* **9**, e017221 (2020). 32757795 10.1161/JAHA.120.017221 PMC7660799

[18] Yang, L. et al. GFRAL is the receptor for GDF15 and is required for the anti-obesity effects of the ligand. *Nat. Med.* **23**, 1158–1166 (2017). 28846099 10.1038/nm.4394

[19] Liss, D. B., Paden, M. S., Schwarz, E. S. & Mullins, M. E. What is the clinical significance of 5-oxoproline (pyroglutamic acid) in high anion gap metabolic acidosis following paracetamol (acetaminophen) exposure? *Clin. Toxicol. (Phila.)* **51**, 817–827 (2013). 24111553 10.3109/15563650.2013.844822

[20] Deng, Y. et al. Upregulated microRNA-381-5p strengthens the effect of dexmedetomidine preconditioning to protect against myocardial ischemia–reperfusion injury in mouse models by inhibiting CHI3L1. *Int. Immunopharmacol.* **92**, 107326 (2021). 33461162 10.1016/j.intimp.2020.107326

[21] van der Ploeg, T., Austin, P. C. & Steyerberg, E. W. Modern modelling techniques are data hungry: a simulation study for predicting dichotomous endpoints. *BMC Med. Res. Methodol.* **14**, 137 (2014). 25532820 10.1186/1471-2288-14-137 PMC4289553

[22] Simell, B. A. et al. Transnational access to large prospective cohorts in Europe: current trends and unmet needs. *N. Biotechnol.* **49**, 98–103 (2019). 30342241 10.1016/j.nbt.2018.10.001

[23] Sajja, A. et al. Comparison of methods to estimate low-density lipoprotein cholesterol in patients with high triglyceride levels. *JAMA Netw. Open* **4**, e2128817 (2021). 34709388 10.1001/jamanetworkopen.2021.28817 PMC8554644

[24] R Core Team. *R: A Language and Environment for Statistical Computing* (R Foundation for Statistical Computing, 2018).

[25] Friedman, J., Hastie, T. & Tibshirani, R. Regularization paths for generalized linear models via coordinate descent. *J. Stat. Softw.* **33**, 1–22 (2010). 20808728 10.18637/jss.v033.i01 PMC2929880

[26] van Buuren, S. & Groothuis-Oudshoorn, K. mice: multivariate imputation by chained equations in R. *J. Stat. Softw.* **45**, 1–67 (2011). 10.18637/jss.v045.i03

[27] Harrell, F. E. Jr. rms: regression modeling strategies. CRAN.R-project.org/package=rms (2020).

[28] Wright, M. N. & Ziegler, A. ranger: a fast implementation of random forests for high dimensional data in C++ and R. *J. Stat. Softw.* **77**, 1–17 (2017). 10.18637/jss.v077.i01

[29] Therneau, T. A package for survival analysis in R. CRAN.R-project.org/package=survival (2022).

[30] White, I. R., Royston, P. & Wood, A. M. Multiple imputation using chained equations: issues and guidance for practice. *Stat. Med.* **30**, 377–399 (2011). 21225900 10.1002/sim.4067

[31] Rubin, D. B. Inference and missing data. *Biometrika* **63**, 581–592 (1976). 10.1093/biomet/63.3.581

[32] Austin, P. C., Harrell, F. E. Jr. & van Klaveren, D. Graphical calibration curves and the integrated calibration index (ICI) for survival models. *Stat. Med.* **39**, 2714–2742 (2020). 32548928 10.1002/sim.8570 PMC7497089

[33] SCORE2 Working Group and ESC Cardiovascular Risk Collaboration. SCORE2 risk prediction algorithms: new models to estimate 10-year risk of cardiovascular disease in Europe. *Eur. Heart J*. **42**, 2439–2454 (2021). 10.1093/eurheartj/ehab309 PMC8248998 34120177

[34] D’Agostino, R. B. Sr., Grundy, S., Sullivan, L. M. & Wilson, P. CHD Risk Prediction Group. Validation of the Framingham coronary heart disease prediction scores: results of a multiple ethnic groups investigation. *JAMA* **286**, 180–187 (2001). 11448281 10.1001/jama.286.2.180

[35] Bergström, G. et al. The Swedish CArdioPulmonary BioImage Study: objectives and design. *J. Intern. Med.* **278**, 645–659 (2015). 26096600 10.1111/joim.12384 PMC4744991

