# Supplementary material for: Markers of imminent myocardial infarction
Source: Nat Cardiovasc Res. 2024 Feb 12;3(2):130–9. doi: 10.1038/s44161-024-00422-2 (PMC11357982; doi:10.1038/s44161-024-00422-2)
Supplement: Supplementary file 1 — Supplementary Notes and References. [file 44161_2024_422_MOESM1_ESM.pdf]

nature cardiovascular research

Letter

https://doi.org/10.1038/s44161-024-00422-2

Markers of imminent myocardial infarction

In the format provided by the authors and unedited

Supplementary Notes

Data Collection
The following section lists documents that describe the initial preparation and data collection.

- Power calculation performed prior to data collection according to data-collection/PowerMIMI_v1_2.pdf.
- Cohort participants defined according to data-collection/MIMI sample definition scheme 1.6, final.pdf and extracted with a script provided centrally (README_v03_01.txt and CHECK_SAMPLE_MIMI_v03_01k4.R).
- Collection and harmonization of baseline covariates in the MIMI cohorts is described in data-collection/cohorts and variables in MIMI.xlsx.

Quality Control (QC)
The following section lists documents that describe some key quality checks performed prior to running the analyses.

Phenotype QC
- **Covariates and outcome**
    - All continuous covariates checked to have reasonable range and distribution.
    - Categorical covariates with expected levels/proportions.
    - Cohorts report time to imminent myocardial infarction (IMI) as MI within 180 or 182 days after baseline. MI events occur uniformly within this follow-up time. Almost all non-cases can be followed for the full 6 months without loss of follow-up according to what the cohorts report. After applying the sampling weights, the Kaplan-Meier survival curves are quite comparable between cohorts. No additional information provided by the cohorts could explain the differences seen.
    - Diagnostic figures of covariates and time-to-event outcome in qc/1.0-20210107-mimi-phenotypes-variables.pdf and qc/1.1-20210107-mimi-phenotypes-survival-by-cohort.pdf.
- **Baseline date (storage time)**
    - Baseline date differs significantly between cohorts from EPIC-CVD (1992-1999) to EpiHealth (2011-2016). Collection date is balanced between cases and non-cases with the exception of Lifelines which unfortunately did not sample cases and non-cases from exactly the same pool of samples due to blood sample availability which resulted in differences in baseline date/storage time for cases and controls.
    - Baseline date by cohort and case status in qc/1.2-20210107-mimi-phenotypes-storage-by-cohort-and-case-status.pdf.

Protein QC
- **Missingness filters**
    - Two sources of missingness are reported by OLINK - technical errors (e.g. a detected air bubble when loading the samples, precipitations that clog the channels) and values below the lower limit of detection (LOD). Reported machine values <LOD will be used.
    - Patients that fail for technical reasons for more than 50% of the plates were excluded. Patients with many <LOD values can simply have many low values for biological reasons and were not excluded. No patient with outlier extreme <LOD missingness was observed.
    - Similar missingness across plates.
    - Distribution of per-sample missingness by missingness type in qc/2.0-20210107-mimi-proteomics-ltlod-sample-miss-step1.pdf and qc/2.1-20210107-mimi-proteomics-qcfail-sample-miss-step1.pdf.
    - Proteins that could be detected in all 6 cohorts and in at least 30 patients in all cohorts combined were included for analysis.

- Proportion of <LOD per-protein missingness was different across panels, with significantly more proteins with a high proportion <LOD on the Immune response, Inflammation, Metabolism, and Organ damage panel as seen in qc/2.2-20210107-mimi-proteomics-miss-by-plate.pdf.
- Heatmap of missingness in the protein matrix (samples = rows, proteins = columns):
- Before filters: qc/2.4-20210107-mimi-proteomics-miss-heatmap-step1.png
    - After per-sample filters: qc/2.5-20210107-mimi-proteomics-mimi-miss-heatmap-step2.png
    - After per-protein filters: qc/2.6-20210107-mimi-proteomics-mimi-miss-heatmap-step3.png

- **Principal components analysis (PCA)**
  - PCA was performed after applying the missingness filters to capture any large scale linear patterns explaining the much variance in the data. Mean-imputation of missing values due to technical reasons (few). Mean-center at 0 and scale to unit variance before PCA.
  - No explanation for top loadings in PCA, shown in qc/2.7-20210107-mimi-proteomics-pc1-2vscohort.pdf. The cohorts samples overlap in PC1 vs PC2, but there is some separation of the cohorts in PC space.
  - PC1 has a very strong correlation with % <LOD. PC2 is not correlated with % <LOD. PCA scatter plot in qc/2.8-20210107-mimi-proteomics-pc1-2vsltlod.pdf.
- **Plate placement**
  - Placement of cases/non-cases as well as cohorts on plates was balanced. Balanced by design, confirmed by those setting up the MIMI study. The EPIC-CVD sub-centers are further spread across multiple plates, but probably not completely at random.
  - Within-cohort plate position unfortunately not random. Strong correlation between baseline date and plate position so it will be hard to separate the effect of these two technical variables. Plate placement by baseline date and cohort in qc/2.9-20210107-mimi-proteomics-storage-time-vs-cohort+plate.pdf.
- **Protein levels vs plate**
  - Olink's plate normalization centers all plates at the same median. The data comes with this normalization which removes much of the plate effect assuming random plate placement. Note that this plate normalization will partially also adjust for storage time/baseline date given the correlation between plate and storage time identified above (incomplete randomization).
  - proteinX ~ plate for each proteinX tested in separate F-tests. The differences in mean protein levels picked up by the F-test are minimal even for the most extreme proteins.
- **Protein levels vs BMI**
  - proteinX ~ bmi + age + sex + cohort with each proteinX tested in separate linear regression models.
  - Established associations between proteins and BMI in previous publications are captured as highly significant with the expected direction of effect. This effect size is quite similar to what previous studies have reported.
    - Positive association with LEP, FABP4 on top.
    - Negative association with IGFBP1, IGFBP2 on top.
    - Top 20 association ranked on a Wald test p-value in qc/3.0-20210107-mimi-proteomics-bmi-top20.pdf
  - The most significant association between BMI and leptin was further tested in analyses stratified by plate and cohort.
    - Per-plate, the results are quite consistent, where all except one per-plate estimate have a confidence interval (CI) that overlaps with the estimate across all plates as shown in qc/3.1-20210107-mimi-proteomics-bmi-vs-leptin-by-plate.pdf.
    - Per cohort, the results for EpiHealth, EPIC-CVD, Estonia, and HUNT are comparable. LifeLines produce a lower than expected point estimate with a wide CI that does not overlap with the estimate across all cohorts. MFM

produces a higher than expected point estimate with a wide CI that does not overlap with the estimate across all cohorts. Everything in the expected direction with p-value below 0.05 for all: qc/3.2-20210107-mimi-proteomics-bmi-vs-leptin-by-cohort.pdf.

- **Proteins on multiple panels**
    - Overall, high correlations of >0.9 in almost all cases: qc/3.3-20210107-mimi-proteomics-protein-duplicates.pdf.

Metabolite QC

- **Missingness filters**
    - Metabolon do not make the distinction between different types of missingness and missing might reflect a technical error, <LOD, or a metabolite not at all present in the sample (true zero). Technical errors are expected to be relatively few according to Metabolon and for some classes of metabolites the most common source of missingness is true zeros.
    - Four problematic samples excluded. Three due to being flagged by Metabolon as problematic and one with a very extreme number of missing metabolites.
    - Sample missingness distribution in qc/4.0-20210107-mimi-metabolomics-sample-miss-step1.pdf and sample missingness by key technical factors in qc/4.1-20210107-mimi-metabolomics-miss-by-sample.pdf where some plates have a clear difference in missingness.
    - Metabolites that could be detected in all 6 cohorts and in at least 30 patients in all cohorts combined were included for analysis.
    - Metabolite missingness by key factors in qc/4.2-20210107-mimi-metabolomics-miss-by-metabolite.pdf where some metabolite categories have metabolites with a very high missingness, e.g. xenobiotics (expected).
    - Heatmap of missingness of the metabolite matrix (samples = rows, proteins = columns):
        - Before filters: qc/4.3-20210107-mimi-metabolomics-miss-heatmap-step1.png
        - After filters: qc/4.4-20210107-mimi-metabolomics-miss-heatmap-step2.png
- **Principal components analysis**
    - Similar setup as for the proteins but missing values for a metabolite were set to min(metabolite). Applied the inverse hyperbolic sine transformation to all metabolites.
    - Top loadings clearly related to use of EDTA/citrate and the EPIC-CVD cohort, shown in qc/5.0-20210107-mimi-metabolomics-pc1-2vscohort.pdf.
    - PC1 has a strong correlation with missingness. PC2 is not correlated with missingness in qc/5.1-20210107-mimi-metabolomics-pc1-2vsltlod.pdf.
- **Citrate vs EDTA**
    - EPIC-CVD is using citrate rather than EDTA. All other cohorts EDTA. There are a few outlier samples and some have both high EDTA and citrate as seen in qc/5.4-20210107-mimi-metabolomics-edta-citrate.pdf.
    - One EPIC-CVD sub-center has a bit higher citrate levels than the other in qc/5.5-20210107-mimi-metabolomics-edta-citrate-in-epic.pdf.
- **Plate placement**
    - Similar plate placement as for the proteins. Plates sent to Metabolon vs Metabolon worksheets are highly correlated. No additional randomization was done at Metabolon. One sample in EPIC-CVD with missing plate for new_worksheet_lcpolar. Not established why.
    - Within-cohort plate position correlated with storage time as for the proteins as seen in qc/5.2-20210107-mimi-metabolomics-storage-time-vs-cohort+plate.pdf

- **Metabolite levels vs plate**
    - metaboliteX ~ plate for each metaboliteX tested in separate F-tests. Some metabolites with a clear plate effect, mostly driven by metabolites with many <LOD/true zero

values at some plates but not at the others as seen in qc/5.6-20210107-mimi-metabolomics-metabolites-with-bonf-sign-plate-effect.pdf.

- **Metabolite levels vs storage time**
    - Fit 1) metaboliteX = intercept, 2) metaboliteX = intercept + spline(storage time). Test if betas spline == 0 in an ANOVA of the nested models. No extreme effect found for any metabolite.
- **Metabolite levels vs BMI**
    - metaboliteX ~ bmi + age + sex + cohort tested for each metaboliteX in separate llinear regression models.
    - Some previously reported associations such as positive association with glutamate, negative association with glycine picked up among the top findings in qc/6.0-20210107-mimi-metabolomics-bmi-top20.pdf.
    - The association between BMI and glutamate was further tested in analyses stratified by plate and cohort.
        - All point estimates in the same direction across cohorts and plates. 95% CI that overlaps with the estimate from all cohorts combined as seen in qc/6.5-20210107-mimi-metabolomics-bmi-vs-glutamate-by-cohort.pdf and qc/6.4-20210107-mimi-metabolomics-bmi-vs-glutamate-by-plate.pdf.
- **Metabolites levels vs sex**
    - sex ~ metaboliteX + age + cohort tested for each metaboliteX in separate Firth's penalized logistic regression models.
    - Several sex hormone metabolites among the top20 results ranked on p-value. Some issues with convergence due to close to perfect separation. Highly significant with very large effect sizes in qc/6.1-20210107-mimi-metabolomics-sex-top20.pdf.
    - The association between 5alpha-androstan-3alpha,17beta-diol disulfate vs sex was further tested in analyses stratified by plate and cohort.
        - Effect estimates are quite comparable across cohorts with being a bit off. Same direction of effect across all plates. For some of the smaller plates the effect size is extreme.
        - qc/6.5-20210107-mimi-metabolomics-bmi-vs-glutamate-by-cohort.pdf and qc/6.4-20210107-mimi-metabolomics-bmi-vs-glutamate-by-plate.pdf.
- **Metabolite levels vs clinical chemistry**
    - High correlation between Metabolon measurements and clinical chemistry measurements for both total cholesterol (TC) and glucose. Above 0.7 unadjusted and above 0.8 adjusting for cohort. Cohort-adjustment has the largest effect for TC, where is a bit off as seen in qc/7.1-20210107-mimi-metabolomics-metabolon-vs-clin-chem-same-metabolite.pdf.

- **Mono/di-acylglycerols vs triacylglycerols**
    - Quite high correlation between TAG (clinical chemistry) and MAGs/DAGs (Metabolon). Especially some abundant DAGs with spearman > 0.7.
    - qc/7.2-20210107-mimi-metabolomics-metabolon-vs-clin-chem-glycerols.pdf
- **Cotinine levels vs smoking**
    - Enrichment of cotinine levels above the detection limit in smokers, followed by previous smokers. Few current smokers without cotinine. Few never smokers with cotinine. Note! cotinine can come from other nicotine-products than cigarettes.
    - qc/7.3-20210107-mimi-metabolomics-cotinine-vs-smoking.pdf

Supplementary References

1. Danesh, J. *et al.* EPIC-Heart: the cardiovascular component of a prospective study of nutritional, lifestyle and biological factors in 520,000 middle- aged participants from 10 European countries. *Eur J Epidemiol* **22**, 129-141 (2007).
2. Langenberg, C. *et al.* Design and cohort description of the InterAct Project: an examination of the interaction of genetic and lifestyle factors on the incidence of type 2 diabetes in the EPIC Study. *Diabetologia* **54**, 2272-2282 (2011).
3. Lind, L. *et al.* EpiHealth: a large population-based cohort study for investigation of gene-lifestyle interactions in the pathogenesis of common diseases. *Eur J Epidemiol* **28**, 189-197 (2013).
4. Leitsalu, L., Alavere, H., Tammesoo, M. L., Leego, E. & Metspalu, A. Linking a population biobank with national health registries-the estonian experience. *J Pers Med* **5**, 96-106 (2015).
5. Leitsalu, L. *et al.* Cohort Profile: Estonian Biobank of the Estonian Genome Center, University of Tartu. *Int J Epidemiol* **44**, 1137-1147 (2015).
6. Krokstad, S. *et al.* Cohort Profile: the HUNT Study, Norway. *Int J Epidemiol* **42**, 968-977 (2013).
7. Scholtens, S. *et al.* Cohort Profile: LifeLines, a three-generation cohort study and biobank. *Int J Epidemiol* **44**, 1172-1180 (2015).
8. Lyssenko, V. *et al.* Clinical risk factors, DNA variants, and the development of type 2 diabetes. *N Engl J Med* **359**, 2220-2232 (2008).
