# Supplementary material for: Markers of imminent myocardial infarction
Source: Nat Cardiovasc Res. 2024 Feb 12;3(2):130–9. doi: 10.1038/s44161-024-00422-2 (PMC11357982; doi:10.1038/s44161-024-00422-2)
Supplement: Supplementary file 2 — Reporting Summary [file 44161_2024_422_MOESM2_ESM.pdf]

nature portfolio

Corresponding author(s): Johan Sundström

Last updated by author(s): Jan 3, 2024

Reporting Summary

Nature Portfolio wishes to improve the reproducibility of the work that we publish. This form provides structure for consistency and transparency in reporting. For further information on Nature Portfolio policies, see our Editorial Policies and the Editorial Policy Checklist.

Statistics

For all statistical analyses, confirm that the following items are present in the figure legend, table legend, main text, or Methods section.

n/a	Confirmed	
☐	☒	The exact sample size (*n*) for each experimental group/condition, given as a discrete number and unit of measurement
☐	☒	A statement on whether measurements were taken from distinct samples or whether the same sample was measured repeatedly
☐	☒	The statistical test(s) used AND whether they are one- or two-sided<br>*Only common tests should be described solely by name; describe more complex techniques in the Methods section.*
☐	☒	A description of all covariates tested
☐	☒	A description of any assumptions or corrections, such as tests of normality and adjustment for multiple comparisons
☐	☒	A full description of the statistical parameters including central tendency (e.g. means) or other basic estimates (e.g. regression coefficient) AND variation (e.g. standard deviation) or associated estimates of uncertainty (e.g. confidence intervals)
☐	☒	For null hypothesis testing, the test statistic (e.g. *F*, *t*, *r*) with confidence intervals, effect sizes, degrees of freedom and *P* value noted<br>*Give P values as exact values whenever suitable.*
☒	☐	For Bayesian analysis, information on the choice of priors and Markov chain Monte Carlo settings
☐	☒	For hierarchical and complex designs, identification of the appropriate level for tests and full reporting of outcomes
☐	☒	Estimates of effect sizes (e.g. Cohen's *d*, Pearson's *r*), indicating how they were calculated

Our web collection on statistics for biologists contains articles on many of the points above.

Software and code

Policy information about availability of computer code

Data collection	No specific software was used.
Data analysis	All analyses were done using R version 4.1.1 with the glmnet, mice, rms, and survival add-on packages. Versions referenced.

For manuscripts utilizing custom algorithms or software that are central to the research but not yet described in published literature, software must be made available to editors and reviewers. We strongly encourage code deposition in a community repository (e.g. GitHub). See the Nature Portfolio guidelines for submitting code & software for further information.

Data

Policy information about availability of data

All manuscripts must include a data availability statement. This statement should provide the following information, where applicable:

- Accession codes, unique identifiers, or web links for publicly available datasets
- A description of any restrictions on data availability
- For clinical datasets or third party data, please ensure that the statement adheres to our policy

Data may be obtained from a third party and are not publicly available. Data accession codes for this study as described below. Researchers can apply to use the Lifelines data used in this study. More information about how to request Lifelines data and the conditions of use can be found on their website (lifelines.nl/researcher/how-to-apply). Transnational access to the European large prospective cohorts was provided by the BBMRI-LPC project funded by the European Commission Seventh Framework Programme [grant no. 313010] (JS). The use of the Estonian Biobank was supported by the European Union through the European

Regional Development Fund (Project No. 2014-2020.4.01.15-0012) and by institutional research funding IUT (IUT20-60) of the Estonian Ministry of Education and Research. We thank Maris Alver (Estonian Biobank) for helping with phenotype data. The use of the Trøndelag Health Study (HUNT) was made possible through a collaboration between HUNT Research Centre (Faculty of Medicine and Health Sciences, Norwegian University of Science and Technology NTNU), Trøndelag County Council, Central Norway Regional Health Authority, and the Norwegian Institute of Public Health. The use of the Lifelines initiative was been made possible by subsidy from the Dutch Ministry of Health, Welfare and Sport, the Dutch Ministry of Economic Affairs, the University Medical Center Groningen (UMCG), Groningen University and the Provinces in the North of the Netherlands (Drenthe, Friesland, Groningen). Lifelines is a multi-disciplinary prospective population-based cohort study examining in a unique three-generation design the health and health-related behaviors of 167,729 persons living in the North of the Netherlands. It employs a broad range of investigative procedures in assessing the biomedical, socio-demographic, behavioral, physical and psychological factors which contribute to the health and disease of the general population, with a special focus on multi-morbidity and complex genetics. The use of EPIC-CVD was supported by funding from the: European Commission Framework Programme 7 (HEALTH-F2-2012-279233), European Research Council (268834), Novartis, UK Medical Research Council (G0800270; MR/L003120/1), British Heart Foundation (SP/09/002; RG/13/13/30194; RG/18/13/33946) and NIHR Cambridge Biomedical Research Centre (BRC-1215-20014) [*]. *The views expressed are those of the author(s) and not necessarily those of the NIHR or the Department of Health and Social Care. The coordination of EPIC is financially supported by International Agency for Research on Cancer (IARC) and also by the Department of Epidemiology and Biostatistics, School of Public Health, Imperial College London which has additional infrastructure support provided by the NIHR Imperial Biomedical Research Centre (BRC). The national cohorts are supported by: Danish Cancer Society (Denmark); Ligue Contre le Cancer, Institut Gustave Roussy, Mutuelle Générale de l'Education Nationale, Institut National de la Santé et de la Recherche Médicale (INSERM) (France); German Cancer Aid, German Cancer Research Center (DKFZ), German Institute of Human Nutrition PotsdamRehbruecke (DIfE), Federal Ministry of Education and Research (BMBF) (Germany); Associazione Italiana per la Ricerca sul Cancro-AIRC-Italy, Compagnia di SanPaolo and National Research Council (Italy); Dutch Ministry of Public Health, Welfare and Sports (VWS), Netherlands Cancer Registry (NKR), LK Research Funds, Dutch Prevention Funds, Dutch ZON (Zorg Onderzoek Nederland), World Cancer Research Fund (WCRF), Statistics Netherlands (The Netherlands); Health Research Fund (FIS) - Instituto de Salud Carlos III (ISCIII), Regional Governments of Andalucía, Asturias, Basque Country, Murcia and Navarra, and the Catalan Institute of Oncology - ICO (Spain); Swedish Cancer Society, Swedish Research Council and County Councils of Skåne and Västerbotten (Sweden); Cancer Research UK (14136 to EPIC-Norfolk; C8221/A29017 to EPIC-Oxford), Medical Research Council (1000143 to EPIC-Norfolk; MR/M012190/1 to EPIC-Oxford). (United Kingdom). We thank all EPIC participants and staff for their contribution to the study, the laboratory teams at the Medical Research Council Epidemiology Unit for sample management and Cambridge Genomic Services for genotyping, Sarah Spackman for data management, and the team at the EPIC-CVD Coordinating Centre for study coordination and administration. The use of SCAPIS was supported by Hjärt-Lungfonden, the Knut and Alice Wallenberg Foundation, the Swedish Research Council and VINNOVA. This research has been conducted using the UK Biobank Resource under Application Number 52678. The computations were enabled by resources in project sens2019006 and sens2020005 provided by the Swedish National Infrastructure for Computing (SNIC) at UPPMAX, partially funded by the Swedish Research Council through grant agreement no. 2018-05973.

Research involving human participants, their data, or biological material

Policy information about studies with human participants or human data. See also policy information about sex, gender (identity/presentation), and sexual orientation and race, ethnicity and racism.

Reporting on sex and gender	Both sexes are included in this study. Data have been sampled and anallysed stratified on sex, to maximise statistical efficiency. Interactions with sex have also been studied, but none found. No data on gender have been collected in this study.
Reporting on race, ethnicity, or other socially relevant groupings	This is a study using existing data from multiple cohorts. Data on multiple socioeconomic variables were sought in all cohorts, but the only socioeconomic variable that could be harmonized between the cohorts was self-reported highest education. No data on race, ethnicity or other socially relevant groupings were available in this study.
Population characteristics	The MIMI study sample draws biobanked blood and data from six Europeans cohorts. Cohort participants with biobanked samples (at least 250 uL of plasma or serum), and who were free from previous clinical cardiovascular disease, were eligible for inclusion in the present study. Exclusion criteria were previous clinical cardiovascular disease, defined as presence at any time before baseline of any of the following: myocardial infarction, coronary procedure, heart failure, structural heart disease, tachyarrhythmias, stroke, thromboembolic disease, and peripheral vascular disease; or renal failure.
Recruitment	Individuals with acute myocardial infarction (ICD-10, I21; ICD-9, 410.0–410.6 and 410.8) as primary cause of hospitalization or death within 6 months after baseline were defined as imminent myocardial infarction (IMI) cases. Up to four cohort representatives per available IMI case were randomly drawn from the full cohort to the subcohort in 50 strata based on sex, age (above/below median), and study center, in a stratified case–cohort design. From the full cohort of 169,053 participants, of which 420 became IMI cases, all 420 IMI cases and 1598 subcohort representatives were drawn.
Ethics oversight	This study in its totality was approved by Uppsala Ethics Authority (Dnr 2016/197). All Estonian Biobank participants have signed a broad informed consent form and the study was carried out under ethical approval 258/M-21 from the Research Ethics Committee of the University of Tartu and data release J08 from the Estonian Biobank. The Lifelines protocol was approved by the UMCG Medical ethical committee under number 2007/152.

Note that full information on the approval of the study protocol must also be provided in the manuscript.

Field-specific reporting

Please select the one below that is the best fit for your research. If you are not sure, read the appropriate sections before making your selection.

☒ Life sciences ☐ Behavioural & social sciences ☐ Ecological, evolutionary & environmental sciences

For a reference copy of the document with all sections, see nature.com/documents/nr-reporting-summary-flat.pdf

Life sciences study design

All studies must disclose on these points even when the disclosure is negative.

Sample size

Sample size calculations are described in supplementary methods, and are available in the supplemental document PowerMIMI_v1_2.pdf. Briefly, in this project a case-cohort analysis will be performed with data from several different cohorts. For each cohort a subcohort of the same size as number of cases will be drawn (stratified on gender and below/above median age). In the analysis, the subcohort and all cases of the cohort (regardless of being drawn in the subcohort or not) will participate. Since the cases will be over represented the observations will be weighted with the inverse probability of being drawn when analysed with Cox regression. We follow the analysis outlined in [1] where cases outside the subcohort contributes to the risk set only at their event times. For each parameter set we will simulate data and for m = 1000 times sample subcohorts and analyse the sampled data set according to [1]. The power is estimated as the proportion of the simulated data sets where the null hypothesis is rejected. We assume there is a contious variable x with a specific HR. We create a large dataset (n=1.000.000) with event time Ti based on x and independent censoring time Ci, i = 1, 2, . . . , 1000000. The observed time for each individual is min(Ti,Ci) with indicator variable = 1 if Ti ≤ Ci and 0 (censored) if Ti > Ci. Since we only observe the cases until day 180, we adjust the times and indicator to that time. When drawing subcohorts to the analysis in the MIMI project a gender and age stratification will be performed. We assume therefore that the gender and age distribution does not matter in this simulation study. The simulation algorithm is the following:
Draw a large data set (n=1.000.000) using
1. x ? N(0,1)
2. T ? Weibull(shape = 1, scale = 9125 × exp(−βx)) where β is log(HR) (corresponds to constant hazard)
3. C ? Weibull(shape = 1, scale = 150)
4. Tobs =min(T,C), δ=1 if T≤C otherwise 0
5. if Tobs>180 set Tobs=180 and δ=0
For m = 1.000 simulations
1. for each cohort, draw number of events and number of eligible individuals according to table 1 from the created data set
2. draw a subsample of size k × number of events from the cohort data, keep track of x variables and sample probabilities
3. analyse the data

[1] Kulathinal, S. et al, Case-Cohort design in practice - experiences from the MORGAM Project Epidemiologic Perspectives and Innovations, 2007, 4:15. 1

Data exclusions

Exclusion criteria were pre-specified to the extent it was possible. Samples that did not satisfy quality-control criteria were initially excluded: exclusion filters were applied separately for the proteomics and metabolomics analyses, and only samples passing quality control for both analyses were included in the analysis set. For the proteomics analysis, samples with more than 50% of panels failing for technical reasons were excluded (n-excluded=33). For the metabolomics analysis, samples were excluded because of low volume, or detection of fewer metabolites than expected (n-excluded=4). Consequently, samples for 420 cases and 1598 subcohort representatives remained for analysis. Next, biomarkers with an extremely high proportion of non-detectable/below LOD measurements were excluded, with the same exclusion filters for proteins and metabolites. Biomarkers had to be detected in all 6 cohorts with at least 30 detectable values across all cohorts (~1·5% of the MIMI samples), or were otherwise excluded.

Replication

The study sample was divided into a discovery sample (EpiHealth, HUNT, and LifeLines; 70% of the sample) and an external validation sample (EPIC-CVD, Estonia, and MFM; 30% of the sample). Considering the limited sample size of the study, an internal validation was performed as an exploratory analysis by randomly splitting the study sample into 70/30 discovery/validation sample, repeated in 100 random draws. Associations of proteins, metabolites, and clinical variables with the risk of subsequent first myocardial infarction within 6 months of baseline in the discovery sample were investigated. Biomarkers that passed multiple-testing bounds were verified in the validation sample (this was done in the external and internal validation sets). Promising markers were thereafter investigated in further models, and their associations with coronary calcium score at a cardiac computer tomography examination were examined in an external population-based cohort. Finally, the possibility to develop a clinical risk prediction algorithm in the discovery sample was investigated, and tested in the validation sample. All replication results described in the manuscript.

Randomization

Replicating biomarkers in the model adjusted for age and sex were subjected to causal assumptions, and a bias-minimized model for each biomarker was investigated, estimating the total effects (including the effects of mediators).

Blinding

Blinding irrelevant; all data were collected before biomarker analyses were made. All biomarker analyses were automated.

Reporting for specific materials, systems and methods

We require information from authors about some types of materials, experimental systems and methods used in many studies. Here, indicate whether each material, system or method listed is relevant to your study. If you are not sure if a list item applies to your research, read the appropriate section before selecting a response.

Materials & experimental systems

n/a	Involved in the study
☒	☐ Antibodies
☒	☐ Eukaryotic cell lines
☒	☐ Palaeontology and archaeology
☒	☐ Animals and other organisms
☒	☐ Clinical data
☒	☐ Dual use research of concern
☒	☐ Plants

Methods

n/a	Involved in the study
☒	☐ ChIP-seq
☒	☐ Flow cytometry
☒	☐ MRI-based neuroimaging
